# Quality of life in cancer patients undergoing anticoagulant treatment with LMWH for venous thromboembolism: the QUAVITEC study on behalf of the Groupe Francophone Thrombose et Cancer (GFTC)

**DOI:** 10.18632/oncotarget.25454

**Published:** 2018-06-05

**Authors:** Dominique Farge, Francis Cajfinger, Nicolas Falvo, Toufek Berremili, Francis Couturaud, Okba Bensaoula, Lionel Védrine, Hocine Bensalha, Isabelle Bonnet, Denis Péré-Vergé, Marie Coudurier, Veronique Li, Hanadi Rafii, Ilham Benzidia, Jean M. Connors, Matthieu Resche-Rigon

**Affiliations:** ^1^ Assistance Publique-Hopitaux de Paris, Saint-Louis Hospital, Internal Medicine, Autoimmune and Vascular Disease Unit, UF 04, Diderot University, Paris, France; ^2^ Medical Oncology, Hôpital Pitié-Salpêtrière, Paris, France; ^3^ Département de Pathologie Vasculaire, CHU Dijon, Dijon Cedex, France; ^4^ Department of Cardiology, Annecy Hospital, Annecy, France; ^5^ Brest University Hospital, CHU de Brest, Brest, France; ^6^ Department of Oncology, CLCC Curie Institute, Centre Rene Huguenin, Saint Cloud, France; ^7^ Hôpital d'Instruction des Armées du Val-de-Grâce, Paris, France; ^8^ Department of Oncology, Hospital of Valenciennes, Valenciennes, France; ^9^ CH Saint Joseph Saint Luc, Lyon, France; ^10^ Chambéry CH, Chambéry, France; ^11^ CH Thonon-Les-Bains, Thonon-les-Bains, France; ^12^ Hematology Division, Harvard Medical School, Boston, MA, USA; ^13^ Service de Biostatistique et Information Médicale, AP-HP Hôpital Saint-Louis, Paris, France

**Keywords:** cancer thrombosis, venous thromboembolism, anticoagulation therapy, quality of life, LMWH

## Abstract

**Background:**

Clinical guidelines recommend at least 3-months low molecular weight heparin (LMWH) treatment for established venous thromboembolism (VTE) in cancer patients. However, no study has analyzed the impact of 3–6 months of LMWH therapy on quality-of-life (QoL) in cancer patients.

**Results:**

Among 400 cancer patients included at M0, 88.8% received long-term LMWH. Using a random-effects linear regression model with time as covariate, QoL scores in the MOS SF-36 (Global HRQoL, 1.3-fold per month [95% confidence interval (CI) 0.81–1.79], *p* < 0.0001) and EORTC QLQ-C30 (global health status/qol, 2.25-fold per month [95% CI 1.63–2.88]; *p* < 0.0001) questionnaires significantly improved over the 6-month study period in patients treated with LMWH, while VEINES-QOL scores did not change. In the MOS SF-36 and EORTC QLQ-C30, the following factors were associated with change in QoL: symptomatic VTE, cancer dissemination and histological type. Factors pertaining to reduced mobility were also identified as significant predictors of QoL outcomes, including being bedridden in the MOS SF-36 and ECOG score ≥ 2 in the EORTC QLQ-C30. Presence of acute infection and not undergoing anti-angiogenic therapy were additional factors associated with QoL improvement in the EORTC QLQ-C30.

**Methods:**

QUAVITEC, a prospective, longitudinal, multicenter study, recruited all consecutive eligible adult cancer patients with objectively confirmed VTE between February 2011 and 2012. Patients were asked to answer three QoL questionnaires at anticoagulant treatment initiation (M0) and at 3 (M3) and 6 (M6)-month follow-ups.

**Conclusion:**

QUAVITEC is the first study to show that QoL was improved in cancer patients receiving long-term LMWH treatment for established VTE.

## INTRODUCTION

Cancer is a major risk factor for venous thromboembolism (VTE) [1], and cancer patients who develop VTE tend to have a poorer prognosis and diminished life expectancy [1]. These patients are also at increased risk of experiencing VTE recurrence and major bleeding complications [1]. Overall, VTE is the second leading cause of death in cancer patients in both medical and surgical settings, after metastasis [1]. Current national and international clinical practice guidelines for the management of VTE in patients with cancer recommend administering LMWH for at least 3 months [2–4]. After 3–6 months, the decision to continue or terminate anticoagulant treatment should be based on the benefit-to-risk ratio, tolerability, drug availability, patient preference, and cancer activity. These recommendations are based on evidence that LMWH is the safest and most efficacious anticoagulant for the treatment and prophylaxis of VTE in this patient population, and that it is associated with a lower incidence of bleeds and recurrent VTE compared to unfractionated heparin (UFH) or vitamin K antagonists (VKAs) [5–7].

There is discrepancy, however, between evidence-based recommendations and clinical practice [8]. The French CARMEN study, which assessed 500 cancer patients treated for VTE two years after the release of French national guidelines, indicated that while physicians administered appropriate anticoagulation for established VTE during the initial phase of treatment (first 10 days) in 98% of cases, guideline adherence dropped to 62% during the treatment maintenance phase (10 days-3 months) [9]. Similarly, an analysis of data from the RIETE registry reported that only 66% of cancer patients were maintained on LMWH for the appropriate recommended treatment duration [10]. Results from a recent study of insurance claims records in the United states indicated that LMWH continues to be under-prescribed in cancer patients; VKA was the most commonly used anticoagulant (50%), with LMWH prescribed in 40% of cases [11]. Similar recent studies carried out on a global scale suggest that LMWH may be prescribed in as few as 25% of cases worldwide [12]. These continued failures in guideline adherence appear to stem in part from 1) concerns about whether cancer patients can tolerate long-term LMWH treatment and their potential side effects, despite their grade A evidence-based established benefit, [2] 2) beliefs about patients’ lack of willingness to accept treatment regimens involving daily subcutaneous injections [13], and 3) preconceptions about the overall impact LMWH treatment will have on QoL. Potential complications of LMWH treatment include heparin-induced thrombocytopenia (HIT), allergic reactions, pain and ecchymosis at injection sites. A number of prospective studies examining long-term use of LMWH in cancer patients have not reported any cases of severe thrombocytopenia and, overall, the incidences of thrombocytopenia in patients on long-term LMWH or receiving VKAs are similar [5–7]. Risk of allergic reaction was not found to differ between LMWH and placebo arms in the MALT study [14]. Pain and development of indurations at injection sites can develop in 30–90% of patients [15], which could prove to be a barrier to treatment compliance. Health-related Quality of life (QoL) has never been assessed in this patient population, although it is an important variable in evaluating treatment effectiveness and compliance in oncology [16, 17]. We therefore designed this prospective, longitudinal, multicenter, observational study to assess QoL in cancer patients initiated on long-term anticoagulant therapy for VTE in a real-world setting with treatment management determined by the local attending physicians.

## RESULTS

**Figure 1:**
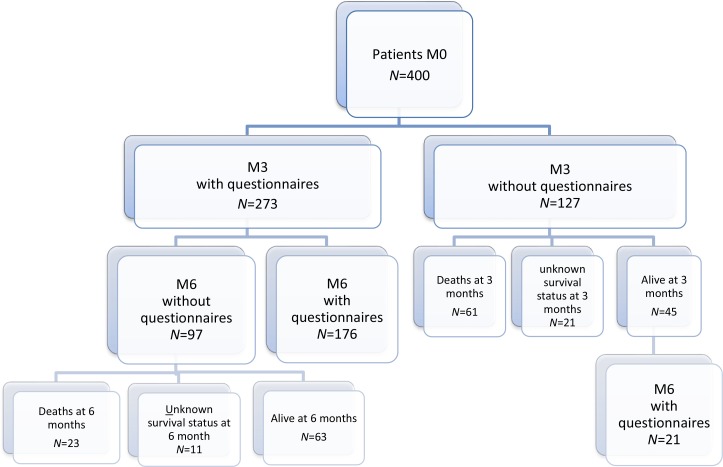
Patient flow chart Flow chart depicts the distribution of completed questionnaires at inclusion, and 3- and 6-month follow-ups.

**Figure 2:**
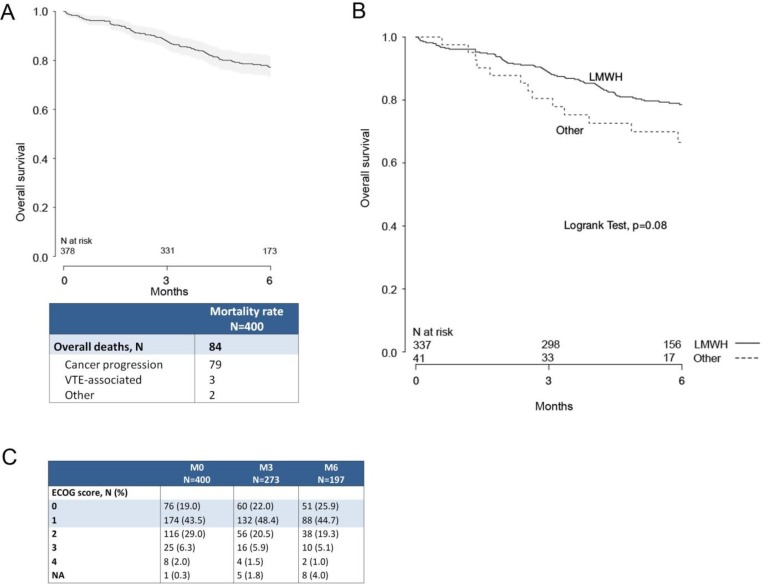
Survival at 3 and 6 months after initiation of anticoagulant therapy (**A**) Kaplan–Meier survival plot of the entire patient population. Only 378 patients were considered in the survival analysis because vital status was not available for twenty-one patients and date of death was not available for one patient. (**B**) Kaplan–Meier survival plot of LMWH group (*n* = 337) versus a pooled group of patients receiving other anticoagulant therapies (*n* = 41). Patients receiving LMWH had numerically better survival outcomes (log-rank test, *p* = 0.08). (**C**) Distribution of ECOG scores at inclusion, 3-month, and 6-month follow-ups.

400 consecutive cancer patients at participating GFTC centers, diagnosed with a VTE and put on an anticoagulant, were included in the study (Figure 1, patient flow chart). At inclusion, 88.8% (355/400) of patients were treated with LMWH, 5.5% (22/400) received a vitamin K antagonist (VKA), 1.5% (6/400) an unfractionated heparin (UFH), 3.75% (15/400) an anti-Xa direct oral anticoagulant, and 0.5% (2/400) another anticoagulant therapy. The overall number of deaths at 6 months was 84, with a mortality rate of 23.7% (95% CI 19.1%–28.1%) (Figure 2A). Seventy-nine deaths were attributed to cancer progression, 3 to VTE, and 2 to others causes (Figure 2A). Kaplan–Meier survival analysis indicated that patients on LMWH (*n* =337) had numerically better survival outcomes relative to other treatments (*n* = 41), but these were not significant (*p* = 0.08 by log-rank test) (Figure 2B). Only 378 patients were considered in the survival analysis because vital status was not available for twenty-one patients and date of death was not available for one patient (Figure 1).

Among the 355 patients given LMWH, 59.4% had metastatic disease (181/355) and 67.1% were on chemotherapy (237/355). Baseline patient characteristics are provided in Table 1 and Supplementary Table 1. During treatment follow-up, 18.9% (67/355) of patients on LMWH reported at least one side effect of injections (number of reports: pain at injection site, 26 (7.3%); ecchymosis, 57 (16.1%); pruritis, 2(0.6%); nodules, 28 (7.9%)).

**Table 1:** Baseline patient characteristics at time of VTE diagnosis for participants receiving LMWH

	LMWH
**Age (years, SD)**	63 (14)
**Female (*N,* %)**	183 (51.6)
**Weight (kg, SD)**	70 (15)
**Height (cm, SD)**	170 (9)
**VTE (*N,* %)** Symptomatic Asymptomatic NA	266 (75.4) 87 (24.7) 2
**Type of VTE (*N,* %)** Superficial vein thrombosis Deep vein thrombosis (DVT) Bilateral thrombosis Pulmonary embolism Central venous catheter-related thrombosis	19 (5.4) 253 (71.3) 20 (5.6) 117 (33.0) 29 (8.2)
**Cancer dissemination (*N,* %)** Local Regional Metastatic NA	52 (17.1) 72 (23.6) 181 (59.3) 50
**Histology (*N,* %)** Adenocarcinoma Squamous Undifferentiated Neuroendocrine Sarcoma Other	223 (62.8) 18 (5.1) 4 (1.1) 5 (1.4) 4 (1.1) 101 (28.5)
**Cancer treatment-related risk factors (*N,* %)** Surgery < 1 month Radiotherapy < 1 month Hormone therapy Tyrosine kinase inhibitors Chemotherapy Anti-angiogenic	36 (10.2) 26 (7.4) 16 (4.5) 8 (2.3) 237 (67.1) 19 (5.4)
**Risk factors (*N,* %)** Prior DVT risk Prior PE risk Obesity (BMI > 35 kg/m^2^) Acute infection Surgery < 3 months Bedridden Varicose veins inferior (arms) Central venous catheter Heart failure Acute respiratory failure Thrombophilia	45 (12.7) 10 (2.8) 11 (3.1) 27 (7.7) 75 (21.3) 51 (14.5) 36 (10.3) 202 (56.9) 4 (1.1) 9 (2.6) 1 (0.3)

Descriptive baseline statistics. Categorical variables are reported as frequencies with percentages. Continuous variables are reported as means with standard deviations (SD).

### Improvement in QoL over the 6-month observation period

Three standardized questionnaires, all previously translated and comprehensively validated into different languages, including French [18–23], were used to measure generic and disease-specific QoL (Supplementary Materials).

**Figure 3:**
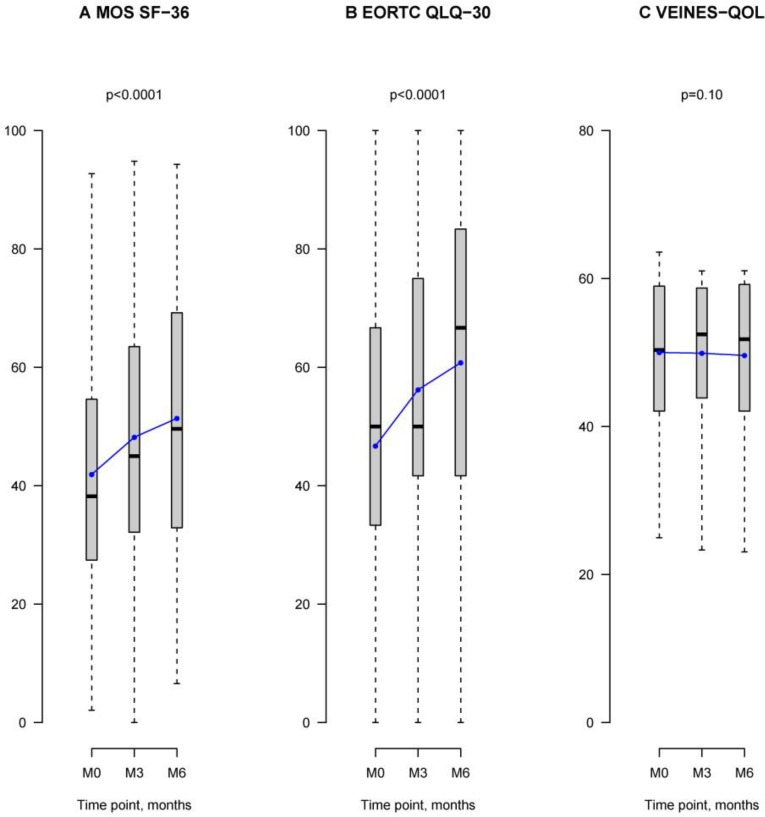
QoL assessments at 0, 3, and 6-month follow-ups in patients treated with LMWH Grey box-and-whisker plots show the median, interquartile, and overall spread of all the QoL data at M0, M3, and M6. Blue linear plots illustrate the mean of the distribution of QoL scores at each observation period M0, M3, and M6. **(A**) Graph illustrates health-related quality of life (HRQoL) scores in the MOS SF-36 survey. At M0, HRQoL mean score was 42 with a median score of 38 [27; 55]. At M3, the mean score was 48 with a median of 45 [32; 63]. At M6, the mean score was 51 with a median of 50 [33; 69]. HRQoL scores increased significantly over the 6-month observation period (*P* < 0.0001). **(B**) Graph shows global health status/QoL scores in the EORTC QLQ-C30 questionnaire which assessed cancer-related QoL. At M0, the mean QoL score was 47, with a median of 50 [33; 67]. At M3, the mean QoL score was 56, with a median of 50 [42; 75]. At M6, the mean score was 61, with a median of 67 [44; 83]. Global health status/QoL scores significantly changed over the 6-month observation period (*P* < 0.0001). **(C**) Graph shows VEINES-QOL scores. At M0, the mean VEINES-QOL score was 50, with median of 50 [42; 59]. At M3, the mean VEINES-QOL score was 50, with a median of 52 [44; 59]. At M6, the mean of VEINES-QOL scores was 50, with a median of 52 [42; 59]. VEINES-QOL scores did not change during the 6-month LMWH treatment period.

In the *Medical Outcome Study 36-item Short-Form Health Survey (MOS SF-36),* [18] global health-related quality of life (HRQoL) scores significantly increased 1.3-fold [95% CI 0.81–1.79] per month over the 6-month observation period (*P* < 0.0001) (Table 2, Figure 3). Improvements in both the physical (1.32-fold [95% CI 0.80–1.84]; *P* < 0.0001) and mental (1.33-fold [95% CI 0.79–1.87]; *P* < 0.0001) health summary scores were observed, as well as across all specific dimensions examined (Table 2). In the patients who answered the questionnaires at 3-month follow-up, there was a mean 2.9 (19) point increase in global HRQoL scores compared to their respective scores at baseline, with a 2.7 (20) point increase in the physical health status summary score, and a 3.1 (22) point increase in the mental health status summary score (Supplementary Table 2). At 6 month follow-up, patients on LMWH showed a mean 7.6 (22) point increase relative to their respective scores at M0, with increases in both the physical (7.7 (23)) and mental health status (7.6 (24)) summary scores (Supplementary Table 2).

**Table 2:** QoL assessments at 0, 3 and 6-month follow-ups in patients treated with LMWH, mean (SD)

	M0		M3		M6			
	*N*	Mean (SD)	*N*	Mean (SD)	*N*	Mean (SD)	Slope of change per month (95%CI)	*P*-value
	355		252		181			
**MOS SF-36**								
Global HRQoL Physical Health summary score Mental Health summary score Physical functioning Physical roles limitation Bodily pain General health Vitality Social functioning Emotional functioning Mental health	355 355 355 355 355 355 355 355 355 355 355	42 (20) 38 (19) 45 (23) 47 (31) 20 (33) 54 (30) 32 (8.3) 37 (22) 55 (29) 32 (42) 58 (21)	252 252 252 252 252 252 252 252 252 252 252	48 (20) 44 (20) 52 (22) 54 (28) 28 (39) 62 (28) 34 (9) 44 (22) 63 (26) 38 (45) 63 (19)	181 181 181 181 181 181 181 181 181 181 181	51 (22) 48 (22) 55 (24) 56 (29) 35 (42) 66 (29) 35 (10) 49 (22) 63 (28) 41 (45) 65 (21)	1.30 (0.81; 1.79) 1.32 (0.80; 1.84) 1.33 (0.79; 1.87) 0.98 (0.31; 1.66) 2.35 (1.37; 3.34) 1.64 (0.87; 2.41) 0.54 (0.29; 0.78) 1.62 (1.10; 2.14) 1.33 (0.63; 2.03) 1.52 (0.43; 2.62) 1.07 (0.60; 1.53)	<0.0001 <0.0001 <0.0001 0.005 <0.0001 <0.0001 <0.0001 <0.0001 0.0002 0.007 <0.0001
**EORTC QLQ-C30**								
Global health status /QOL Physical functioning Role functioning Emotional functioning Cognitive functioning Social functioning Fatigue Nausea and vomiting Pain Dyspnea Insomnia Appetite loss Constipation Diarrhea Financial difficulties	351 348 343 353 352 351 344 346 354 343 342 345 350 351 349	47 (24) 66 (27) 53 (37) 67 (26) 76 (26) 60 (32) 55 (31) 17 (26) 35 (33) 36 (37) 39 (36) 38 (39) 26 (33) 21 (31) 12 (25)	250 249 248 250 250 250 249 249 249 246 246 248 248 249 248	56 (23) 72 (22) 64 (33) 73 (24) 82 (21) 69 (29) 43 (28) 13 (24) 26 (27) 28 (33) 28 (31) 24 (33) 18 (25) 18 (28) 10 (23)	178 178 178 179 179 179 176 177 180 177 176 177 178 176 178	61 (24) 72 (24) 66 (33) 77 (23) 82 (24) 74 (30) 36 (29) 9.3 (19) 24 (29) 23 (28) 25 (30) 19 (29) 18 (28) 15 (26) 7.9 (21)	2.25 (1.63; 2.88) 0.74 (0.12; 1.37) 1.93 (1.03; 2.84) 1.51 (0.93; 2.10) 0.87 (0.31; 1.42) 1.99 (1.18; 2.79) –2.78 (–3.53; –2.03) –1.11 (–1.75; –0.47) –1.68 (–2.49; –0.87) –1.92 (–2.68; –1.15) –2.26 (–3.15; –1.38) –3.12 (–4.04; –2.21) –1.39 (–2.15; –0.62) –0.91 (–1.70; –0.12) –0.55 (–1.02; –0.08)	<0.0001 0.02 <0.0001 <0.0001 0.002 <0.0001 <0.0001 0.0008 <0.0001 <0.0001 <0.0001 <0.0001 0.00041 0.02 0.02
**Veines-QOL**								
VEINES-QOL VEINES-Sym	340 344	50 (10) 50 (10)	240 241	50 (10) 50 (10)	168 169	50 (10) 50 (10)	–0.20 (–0.43; 0.04) –0.08 (–0.33; 0.16)	0.10 0.52

Random-effects linear regression model with time as a covariate was performed on the QoL outcomes in the three questionnaires (MOS SF-36, EORTC QLQ-C30, VEINES-QOL). Estimated change in QoL scores per month (95% confidence Intervals) and corresponding *p*-values are provided.

In the *European Organization for Research and Treatment of Cancer Quality of Life Questionnaire* (EORTC QLQ-C30cancer-related QoL survey, global Health status/QoL significantly increased 2.25-fold [95% CI 1.63–2.88] per month over the 6-month study period (*P* < 0.0001) (Table 2, Figure 3). Underlying improvements across all *functioning* and *symptom* scales of the EORTC QLQ-C30 survey were observed (Table 2). Compared to scores at M0, global Health status/QoL scores increased by a mean 6.6 (26) points at 3-month follow-up, and by 11 (29) points at 6-month follow-up (Supplementary Table 2).

In the *Venous Insufficiency Epidemiological and Economic Study (VEINES)-QOL questionnaire*, [21–23] scores did not significantly change during the study (-0.20-fold [95% CI–0.43–0.04]; *p* = 0.10) (Table 2). This was also the case in the symptom-specific scale of the questionnaire, VEINES-Sym (-0.08-fold [95% CI–0.33–0.16]; *p* = 0.52).

### Predictors of QoL outcomes in cancer patients with established VTE receiving Long-term LMWH

Independent predictors of QoL outcomes in the MOS SF-36 and the EORTC QLQ-C30 questionnaires during the study period were identified (Supplementary Tables 3–5). In the MOS SF-36 survey, decreased functional activity at inclusion (ie.: bedridden; = 0.016) and symptomatic as opposed to asymptomatic VTE (*P* < 0.001) were independent predictors of improvement in HRQoL scores (Supplementary Tables 3, 4). Cancer dissemination (local, regional, metastatic; *p* = 0.022) and cancer histological type (adenocarcinoma, squamous, neuroendocrine, sarcoma, other; *p* = 0.001) were also predictors of QoL outcomes (Supplementary Tables 3, 4).

In the EORTC QLQ-C30, presence of the following factors at inclusion were independent predictors of improvement in global Health status/QoL scores over the 6-month study period: decreased functional activity measured by ECOG scores (ie.: 2, 3, 4 versus 0, 1 score, *p* = 0.011), symptomatic as opposed to asymptomatic VTE (*p* = 0.045), presence of acute infection (*p* = 0.033), and not undergoing anti-angiogenic therapy (*p* = 0.039) (Supplementary Tables 3 and 5). Cancer dissemination (*p* = 0.044) and cancer histology (*p* = 0.002) were also associated with QoL outcomes in the cancer-specific EORTC QLQ-C30 (Supplementary Tables 3, 5).

## DISCUSSION

QUAVITEC is the first study to assess HRQoL in cancer patients with acute VTE undergoing anticoagulant treatment over six months. In this independent prospective observational study, QoL was assessed in a real-world setting with anticoagulant therapy determined by expert treating clinicians who were members of the *Groupe Francophone Thrombose et Cancer* (GFTC), which aims to improve good clinical practices for the treatment of VTE in cancer patients. The majority of patients were treated with LMWH (88.8%), consistent with evidence-based clinical practice guidelines. In the MOS SF-36 questionnaire, significant improvements in HRQoL were observed over the 6-month study period in patients receiving LMWH, relative to their respective scores at inclusion. The EORTC QLQ-C30 survey similarly indicated a robust significant increase in cancer-related global health status/QoL over time in patients on LMWH. Studies estimating the minimal important differences (MID) in QoL scores suggest that the significant increases in QoL observed here in the MOS SF-36 and EORTC QLQ-C30 questionnaires are clinically meaningful. Studies in cancer patients have estimated that the MID in the EORTC QLQ-C30 questionnaire to range between 6 and 15 points [17, 24, 25] and that statistically significant changes in scores that are less than 6 are unlikely to be of clinical significance for patients [26]. In the MOS SF-36, the physical and mental health summary scores we observed also exceeded reported estimated MID (3–5 points) [27]. Scores in the disease-specific VEINES-QOL survey, which assesses QoL and symptom burden in patients with DVT, did not change during the 6-month study observation period. The Kaplan–Meier survival analysis indicated that patients treated with LMWH had non-significant better survival outcomes than patients receiving other anticoagulant therapies. However, the number of patients receiving treatments other than LMWH were few (*n =* 41). Furthermore, these data are difficult to interpret because the patients on other treatments, were patients in whom LMWH was contra-indicated and therefore likely to be more medically ill, such as hospitalized patients on iv unfractionated heparin, or patients with severe renal insufficiency on warfarin.

ECOG scores indicate that at follow-up the surviving study population had shifted toward improved health, with the percentage of patients with ECOG scores of 0 or 1 increasing from 62.5% at M0 to 70.6% at M6 (Figure 2C). The observation that QoL improved with increasing ECOG scores over the six-month study period suggests that the LMWH treatment regimen did not have a negative impact on QoL. In our study, 18.9% of patients on LMWH reported at least one side effect of injection, and 7.3% reported experiencing pain associated with LMWH injection. There is the possibility that painful side effects associated with daily LMWH injections may have an impact on treatment compliance in certain patients. However, an international qualitative survey of anticoagulant healthcare providers and their patients reported that physicians underestimate patient willingness to follow long-term treatment regimens that require injection, when it is the optimal treatment choice [13]. A later study assessed which features of anticoagulant treatment are most important to patients in making anticoagulant treatment decisions. Patients reported that avoiding interference with their cancer treatment was the most important factor (39%). This was followed by anticoagulant safety and efficacy (ie.: low thrombosis rate (24%) and minimizing bleed risk (19%)), which were rated to be of greater importance over convenience of route of administration (oral over injection, 13%) [28]. In the TROPIQUE study, patients reported that long-term LMWH was very convenient (mean score, 79.7 (SD:17.1)), and treatment satisfaction was relatively high (62.9 (SD:16.7)), particularly on measures of reassurance about treatment efficacy and experience with side effects. However, 23.4% of patients did report being unsatisfied or very unsatisfied with daily LMWH injections, indicating that a subpopulation of patients may significantly benefit from oral anticoagulant treatment as appropriate options become available. Studies in non-cancer patients are confirming that DOACs are associated with improved QoL compared to LMWH and VKA [29]. Although DOACS may be effective and safe in cancer patients, dedicated studies are needed before DOACS can be used with certainty in the cancer patient population. At present, LMWH is the appropriate first-line choice for the majority of cancer patients with VTE [2]. Our results indicate that HRQoL, as assessed by the MOS SF-36, EORTC QLQ-C30, and VEINES-QOL surveys, is not negatively affected by the use of LMWH; HRQoL increased in surviving patients despite LMWH treatment.

Our study had a number of limitations. This independent prospective observational real-life study could not be randomized due to the fact that this patient population required anticoagulant treatment with LMWH, unless contra-indicated, according to evidence-based clinical practice guidelines. While the drop-out rate was relatively low (32 of 400), a significant proportion of patients died over the 6-month study period, mainly due to cancer progression (23.7%). Notably, the mortality rate observed in our study was similar to the rates reported in the CLOT and CATCH trials, which also had 6-month follow-ups. ECOG scores improved in the group of surviving patients over the study period. Thus, QoL improved with ECOG scores, suggesting that LMWH did not hinder an increased QoL associated with a better global health.

## MATERIALS AND METHODS

### Patients

In this prospective, longitudinal, observational multicenter study, all consecutive cancer patients (>18 years), objectively diagnosed with symptomatic or asymptomatic deep vein thrombosis (DVT), pulmonary embolism (PE), or superficial vein thrombosis, were recruited at 22 participating centers from the Groupe Francophone Thrombose and Cancer (www.thrombose-cancer.com) between February 2011 and 2012. Patients with less than 3 months life-expectancy or with unfeasible follow-up, those incapable of answering the questionnaires or who did not provide written informed consent were excluded from the study. The protocol was approved by the ethics committees of each participating center, according to local laws.

### Procedures

The study primary objective was to evaluate QoL in cancer patients at the time of VTE diagnosis and start of anticoagulant therapy (M0), after 3 (M3) and 6 (M6) months of anticoagulant treatment for objectively confirmed VTE. Secondary objectives were survival after 3 and 6 months of anticoagulant therapy. Anticoagulant treatment was determined by the attending physicians, based on patient characteristics and comorbidities, and consistent with evidence-based clinical practice guidelines, most patients were under long-term LMWH, unless contraindicated.

Three standardized questionnaires, all previously translated and comprehensively validated into different languages, including French [18–23], were used to measure generic and disease-specific QoL (Supplementary Materials).

In the *Medical Outcome Study 36-item Short-Form Health Survey (MOS SF-36)*, [18], for generic Health-Related Quality of Life (HRQoL), generates 8 subscales (from scores of 0 to 100) and two summary scores that measure physical health (physical component score (PCS)) and mental health (mental component score (MCS)) status. The *European Organization for Research and Treatment of Cancer Quality of Life Questionnaire* (EORTC QLQ-C30) [20] is used to assess QoL in cancer patients, through 9 multi-item scales that span three dimensions: functional, symptoms and a global health status and quality-of-life scale (HRQoL). The *Venous Insufficiency Epidemiological and Economic Study (VEINES)-QOL questionnaire,* for venous disease-specific QOL, is a 26-item questionnaire measuring the global impact of chronic venous disease [21] or recent DVT [22] on QoL with questions on the intensity and severity (VEINES-Sym subscale) and the impacts of venous symptoms [21, 23].

### Statistical analysis

Categorical variables were reported as frequencies with percentages and continuous variables as means with standard deviations (SD). To assess the impact of time on QoL scores, random-effects linear regression models with time as covariate were used. Patients’ random effects were considered on the intercept and on the time coefficients to take in account the intra-patient correlations. Impact of patient characteristics on change in QoL were assessed using a random-effects linear regression model including a cross-level interaction term for each characteristic and time. Interaction terms for binary characteristics were tested using Wald tests, while interaction terms for non-binary qualitative characteristics were globally tested using likelihood ratio tests. All analyses were performed in the group of patients treated by LWMH only. The Kaplan Meier estimator was used to estimate the probability of survival and the log-rank test to compare survival between LMWH and other treatments. All statistical tests were two-sided and a significance level of α = 0.05 was applied. Analyses were run using R 2.15.2 [30].

## CONCLUSIONS

VTE in patients with cancer has a negative impact on morbidity and survival, and appropriate anticoagulant therapy is crucial for improving health outcomes in these patients. Despite this, studies continue to consistently show that LMWH is under-utilized in cancer patients. One barrier to conforming to evidence-based clinical practice guidelines has been physician concern about the burden of LMWH injections on QoL. Our study findings show that LMWH did not hinder QoL improvements in cancer patients who survived to 6-month follow-up and exhibited increased health overall. Given how crucial anticoagulant treatment is to decreasing morbidity and mortality, these data contribute to dispelling concerns about the negative impact of LMWH treatment regimens on overall patient well-being and QoL.

## SUPPLEMENTARY MATERIALS TABLES


