# Supplementary material for: Quality of life in cancer patients undergoing anticoagulant treatment with LMWH for venous thromboembolism: the QUAVITEC study on behalf of the Groupe Francophone Thrombose et Cancer (GFTC)
Source: Oncotarget. 2018 Jun 5;9(43):26990–9. doi: 10.18632/oncotarget.25454 (PMC6007467; doi:10.18632/oncotarget.25454)
Supplement: Supplementary file 1 [file oncotarget-09-26990-s001.pdf]

Quality of life in cancer patients undergoing anticoagulant treatment with LMWH for venous thromboembolism: the QUAVITEC study on behalf of the Groupe Francophone Thrombose et Cancer (GFTC)

SUPPLEMENTARY MATERIALS

Supplementary Table 1: Baseline patient characteristics at time of VTE diagnosis for participants receiving LMWH –Primary cancer site

	**LMWH**
**Primary Cancer site**	***N*** **(%)**
Breast	54 (15.2)
Lung	45 (12.7)
Colon	35 (9.9)
Ovary	25 (7.0)
Pancreas	24 (6.8)
Non-Hodgkin’s lymphoma	22 (6.2)
Rectum	13 (3.7)
Myeloma	13 (3.7)
Prostate	12 (3.4)
Bladder	12 (3.4)
Stomach	11 (3.1)
Melanoma	10 (2.8)
Testicle	9 (2.5)
Oral, Head and neck	9 (2.5)
Cervix/endometrium	7 (2.0)
Brain	6 (1.7)
Kidney	6 (1.7)
Acute lymphocytic leukemia	6 (1.7)
Hodgkin’s lymphoma	5 (1.4)
Sarcoma	3 (0.9)
Esophagus	3 (0.9)
Acute myelogenous leukemia	2 (0.6)
Liver	2 (0.6)
Carcinoma of unknown origin	2 (0.6)
Other	19 (5.4)

Supplementary Table 2: Differences QoL scores between baseline and 3- or 6-month follow-ups

	N	**M3-M0**	*N*	**M6-M0**
**MOS SF-36**		Mean (SD)		Mean (SD)
Global HRQoL	252	2.9 (19)	181	7.6 (22)
Physical Health summary score	252	2.7 (20)	181	7.7 (23)
Mental Health summary score	252	3.1 (22)	181	7.6 (24)
Physical functioning	252	0.42 (28)	181	4.9 (31)
Physical roles limitation	252	6.2 (39)	181	15 (45)
Bodily pain	252	3.7 (31)	181	8 (36)
General health	252	0.53 (9.8)	181	3.1 (11)
Vitality	252	2.4 (23)	181	8.9 (23)
Social functioning	252	4.2 (29)	181	6.7 (32)
Emotional functioning	252	2.6 (44)	181	9.4 (52)
Mental health	252	3.2 (20)	181	5.5 (21)
**EORTC QLQ-30**				
Global health status /QOL	249	6.6 (26)	176	11 (29)
Physical functioning	245	0.96 (24)	174	3.2 (27)
Role functioning	240	3.8 (40)	172	10 (40)
Emotional functioning	249	4.2 (24)	179	9.2 (27)
Cognitive functioning	249	2.8 (25)	178	3.9 (25)
Social functioning	247	3.8 (33)	178	11 (37)
Fatigue	241	–5.8 (30)	170	–14 (34)
Nausea and vomiting	243	–0.89 (29)	173	–6.2 (30)
Pain	248	–6 (34)	180	–9 (38)
Dyspnea	239	–3.5 (34)	171	–11 (34)
Insomnia	237	–8 (37)	169	–13 (41)
Appetite loss	241	–7.1 (41)	172	–13 (41)
Constipation	245	–6.3 (31)	177	–7 (35)
Diarrhea	246	–1.2 (35)	176	–5.1 (36)
Financial difficulties	245	–1.5 (21)	175	–3.2 (19)
**VEINES-QOL**				
VEINES-QOL	235	–1.4 (9.2)	165	–1.4 (10)
VEINES-Sym	237	–0.77 (10)	164	–0.23 (11)

Supplementary Table 3: Impact of cancer, VTE, and individual patient characteristics on changes in MOS SF-36 HRQoL (A) and EORTC QLQ-C30 (B) scores in participants treated with LMWH

A

	MOS SF-36 HRQoL									
**VARIABLES**	**Change rate per month (slope)**			**Variable of interest**				**Interaction between time and Variable of interest (change in the slope due to the variable)**		
	**Estim.**	**SE**	***P***	**Value**	**Estim.**	**SE**	***P***	**Estim.**	**SE**	***P***
**ECOG**	1.02	0.31	0.001	**-0,1**	1			1		
				**(2,3,4)**	-13.63	2.03	<.001	0.69	0.54	0.202
**Dissemination**	2.33	0.63	<.001	**Local**	1			1		*
				**Regional**	3.46	3.58	0.335	-1.27	0.83	0.128
				**Metastatic**	4.32	3.1	0.165	-1.72	0.73	0.019
**Histology**	0.81	0.32	0.012	**Adenocar.**	1			1		**
				**Squamous**	-4.89	4.79	0.308	0.82	1.14	0.474
				**Neuroendoc.**	-14.11	8.86	0.112	2.48	2.24	0.268
				**Sarcoma**	-4.39	9.87	0.657	0.73	2.39	0.759
				**Other**	-0.08	2.36	0.974	1.33	0.56	0.017
**Cancer surgery**	1.2	0.27	<.001	**No**	1			1		
				**Yes**	0.02	3.44	0.996	1.04	0.86	0.226
**Tyrosine Kinases**	1.34	0.26	<.001	**No**	1			1		
				**Yes**	6.74	6.99	0.336	-2	1.83	0.276
**Chemotherapy**	1.14	0.41	0.006	**No**	1			1		
				**Yes**	-1.98	2.21	0.369	0.24	0.52	0.64
**Anti-angiogenics**	1.36	0.26	<.001	**No**	1			1		
				**Yes**	11.48	4.55	0.012	-0.99	1.04	0.34
**Previous DVT**	0.4	1.36	0.77	**No**	1			1		
				**Yes**	-3.63	3.11	0.244	0.5	0.72	0.491
**Previous PE**	-3.02	2.57	0.24	**No**	1			1		
				**Yes**	-9.86	6.19	0.112	2.21	1.3	0.09
**Obesity**	-0.08	2.82	0.977	**No**	1			1		
**(BMI > 35kg/m2)**				**Yes**	-7.78	5.98	0.194	0.7	1.43	0.625
**Acute Infection**	4.92	1.95	0.012	**No**	1			1		
				**Yes**	9.73	3.89	0.013	-1.85	1	0.066
**Bedridden**	4.76	1.44	0.001	**No**	1			1		
				**Yes**	12.31	2.92	<.001	-1.84	0.76	0.016
**Central venous catheter**	0.12	0.78	0.873	**No**	1			1		
				**Yes**	-4.59	2.14	0.032	0.8	0.52	0.122
**Symptomatic VTE**	-0.45	0.49	0.361	**No**	1			1		
				**Yes**	-7.67	2.39	0.001	2.36	0.57	<.001
**Superficial VTE**	1.2	0.26	<.001	**No**	1			1		
				**Yes**	4.05	4.61	0.381	1.6	1.02	0.118
**Deep VTE**	0.66	0.47	0.162	**No**	1			1		
				**Yes**	-5.53	2.28	0.016	0.89	0.56	0.109
**Bilateral VTE**	1.33	0.26	<.001	**No**	1			1		
				**Yes**	-8.57	4.51	0.058	-0.73	1.2	0.544
**Pulmonary embolism**	1.52	0.3	<.001	**No**	1			1		
				**Yes**	2.3	2.2	0.296	-0.7	0.54	0.192
**CVC-related thrombosis**	1.32	0.26	<.001	**No**	1			1		
				**Yes**	6.86	3.78	0.071	-0.18	0.98	0.856

**P*.value of the global likelihood ratio interaction test : 0.022.

***P*.value of the global likelihood ratio interaction test : 0.001.

B

VARIABLES	Change rate per month (slope)			Variable of interest				Interaction between time and Variable of interest (change in the slope due to the variable)		
	EORTC QLQ-C30									
	Estim.	SE	*P*	Value	Estim.	SE	*P*	Estim.	SE	*P*
**ECOG**	1.58	0.39	<.001	**-0,1**	1			1		
				**(2,3,4)**	-15.08	2.45	<.001	1.71	0.67	0.011
**Dissemination**	3.09	0.81	<.001	**Local**	1			1		***
				**Regional**	2.27	4.24	0.593	-0.77	1.06	0.466
				**Metastatic**	2.14	3.65	0.559	-1.59	0.93	0.089
**Histology**	1.98	0.41	<.001	**Adenocar.**	1			1		****
				**Squamous**	-2.17	5.67	0.703	-0.56	1.47	0.702
				**Neuroendoc.**	-15.81	10.5	0.133	1.34	2.86	0.639
				**Sarcoma**	-2.5	11.69	0.831	0.2	3.05	0.947
				**Other**	2.09	2.81	0.458	0.95	0.71	0.182
**Cancer surgery**	2.2	0.34	<.001	**No**	1			1		
				**Yes**	3.18	4.09	0.437	0.64	1.09	0.559
**Tyrosine Kinases**	2.17	0.32	<.001	**No**	1			1		
				**Yes**	-8.81	8.31	0.29	4.2	2.3	0.068
**Chemotherapy**	2.07	0.52	<.001	**No**	1			1		
				**Yes**	-2.1	2.63	0.425	0.27	0.66	0.679
**Anti-angiogenics**	2.42	0.33	<.001	**No**	1			1		
				**Yes**	6.57	5.45	0.228	-2.72	1.31	0.039
**Previous DVT**	1.63	1.73	0.345	**No**	1			1		
				**Yes**	1.31	3.67	0.722	0.37	0.92	0.687
**Previous PE**	-2.92	3.31	0.379	**No**	1			1		
				**Yes**	-2.78	7.3	0.703	2.67	1.68	0.113
**Obesity**	-0.83	3.54	0.815	**No**	1			1		
**(BMI > 35kg/m2)**				**Yes**	-19.74	7	0.005	1.54	1.79	0.389
**Acute Infection**	7.6	2.49	0.002	**No**	1			1		
				**Yes**	9.67	4.7	0.04	-2.74	1.28	0.033
**Bedridden**	5.13	1.82	0.005	**No**	1			1		
				**Yes**	9.91	3.54	0.005	-1.53	0.96	0.111
**Central venous catheter**	0.67	1	0.501	**No**	1			1		
				**Yes**	-4.98	2.53	0.05	1.14	0.66	0.086
**Symptomatic VTE**	1.19	0.63	0.059	**No**	1			1		
				**Yes**	-6.74	2.86	0.019	1.47	0.73	0.045
**Superficial VTE**	2.15	0.33	<.001	**No**	1			1		
				**Yes**	1.97	5.48	0.72	1.39	1.29	0.285
**Deep VTE**	2.79	0.6	<.001	**No**	1			1		
				**Yes**	-0.43	2.74	0.875	-0.75	0.71	0.291
**Bilateral VTE**	2.29	0.33	<.001	**No**	1			1		
				**Yes**	-4.66	5.38	0.387	-1.03	1.5	0.493
**Pulmonary embolism**	2.06	0.39	<.001	**No**	1			1		
				**Yes**	-2.08	2.63	0.43	0.61	0.69	0.376
**CVC-related thrombosis**	2.33	0.33	<.001	**No**	1			1		
				**Yes**	11.02	4.48	0.014	-0.96	1.24	0.439

*** *P*.value of the global likelihood ratio interaction test : 0.044.
**** *P*.value of the global likelihood ratio interaction test : 0.002.

Supplementary Table 4: Means (SD) of the MOS SF-36 HRQoL scores at M0, M3, M6 according to cancer, VTE, and individual patient characteristics

	M0		M3		M6	
	N	Mean (SD)	*N*	Mean (SD)	*N*	Mean (SD)
	355		252		181	
**ECOG**						
ECOG (0,1)	223	47 (20)	178	51 (21)	120	55 (22)
ECOG (2,3,4)	131	33 (16)	74	42 (18)	60	44 (20)
**Dissemination**						
Local	52	39 (20)	40	46 (20)	29	54 (21)
Regional	72	42 (20)	51	47 (20)	42	49 (21)
Metastatic	181	43 (20)	127	48 (20)	81	50 (23)
**Histology**						
Adenocarcinoma	223	42 (20)	156	48 (20)	109	51 (23)
Squamous	18	36 (23)	15	48 (22)	9	43 (24)
Neuroendocrine	5	32 (7)	4	27 (10)	2	64 (20)
Sarcoma	4	38 (15)	3	39 (6)	2	42 (1)
Other	100	42 (20)	72	51 (20)	57	54 (20)
**Cancer surgery <1 month**						
Yes	36	41 (23)	27	55 (19)	16	51 (24)
No	317	42 (19)	225	47 (20)	164	51 (21)
**Tyrosine Kinases**						
Yes	8	53 (25)	6	38 (30)	3	56 (24)
No	345	42 (20)	246	48 (20)	177	51 (22)
**Chemotherapy**						
Yes	237	41 (19)	163	48 (19)	107	52 (21)
No	116	43 (21)	89	49 (22)	73	49 (23)
**Anti-angiogenics**						
Yes	19	51 (19)	16	59 (19)	12	47 (21)
No	334	41 (20)	236	47 (20)	168	51 (22)
**Previous DVT**						
Yes	45	45 (17)	34	49 (20)	28	52 (23)
No	296	42 (20)	209	48 (20)	145	51 (22)
**Previous PE**						
Yes	10	51 (17)	9	53 (20)	8	47 (25)
No	332	42 (20)	234	48 (20)	165	52 (22)
**Obesity (BMI > 35 kg/m²)**						
Yes	11	49 (29)	9	55 (27)	6	61 (27)
No	335	42 (19)	237	48 (20)	169	51 (22)
**Acute Infection**						
Yes	27	34 (17)	17	42 (18)	12	57 (21)
No	312	43 (20)	223	49 (20)	159	51 (22)
**Bedridden**						
Yes	51	31 (19)	30	46 (24)	23	46 (23)
No	286	44 (19)	210	49 (20)	145	53 (22)
**Central venous catheter**						
Yes	202	44 (20)	142	50 (20)	97	52 (22)
No	142	40 (20)	101	46 (21)	75	50 (21)
**Symptomatic VTE**						
Yes	266	40 (20)	184	48 (20)	135	53 (22)
No	87	48 (20)	66	47 (22)	44	48 (21)
**Superficial VTE**						
Yes	19	46 (23)	13	57 (23)	13	64 (27)
No	336	42 (20)	239	48 (20)	168	50 (21)
**Deep VTE**						
Yes	253	40 (19)	180	47 (20)	130	51 (21)
No	102	46 (22)	72	51 (20)	51	53 (24)
**Bilateral VTE**						
Yes	20	34 (16)	11	41 (20)	8	38 (24)
No	335	42 (20)	241	49 (20)	173	52 (21)
**Pulmonary embolism**						
Yes	117	43 (21)	82	49 (21)	57	51 (23)
No	238	41 (19)	170	48 (20)	124	52 (21)
**CVC-related thrombosis**						
Yes	29	48 (21)	22	55 (19)	11	58 (19)
No	326	41 (20)	230	48 (20)	170	51 (22)

Supplementary Table 5: Means (SD) of the EORTC QLQ-C30 HRQoL scores at M0, M3, M6 according to cancer, VTE, and individual patient characteristics

	M0		M3		M6	
	N	Mean (SD)	*N*	Mean (SD)	*N*	Mean (SD)
	355		252		181	
**ECOG**						
ECOG (0,1)	221	53 (23)	177	57 (23)	119	64 (23)
ECOG (2,3,4)	130	36 (22)	73	53 (23)	58	54 (24)
**Dissemination**						
Local	52	45 (23)	39	56 (25)	29	64 (20)
Regional	70	47 (25)	50	57 (20)	41	60 (24)
Metastatic	180	47 (24)	127	56 (24)	80	57 (25)
**Histology**						
Adenocarcinoma	221	46 (23)	155	56 (24)	106	59 (24)
Squamous	18	44 (23)	15	53 (20)	9	47 (23)
Neuroendocrine	5	35 (12)	4	29 (14)	2	67 (24)
Sarcoma	4	46 (5)	3	47 (17)	2	62 (29)
Other	98	49 (27)	71	59 (21)	57	66 (23)
**Cancer surgery <1 month**						
Yes	36	47 (23)	27	67 (18)	16	58 (20)
No	313	46 (24)	223	55 (23)	161	61 (24)
**Tyrosine Kinases**						
Yes	8	42 (30)	6	50 (33)	3	83 (29)
No	341	47 (24)	244	56 (23)	174	60 (24)
**Chemotherapy**						
Yes	233	46 (23)	161	55 (23)	105	62 (24)
No	116	47 (25)	89	59 (22)	72	58 (23)
**Anti-angiogenics**						
Yes	19	53 (22)	16	53 (20)	12	48 (25)
No	330	46 (24)	234	56 (23)	165	61 (23)
**Previous DVT**						
Yes	45	46 (23)	34	55 (24)	28	58 (24)
No	292	47 (24)	207	56 (23)	142	62 (24)
**Previous PE**						
Yes	10	52 (25)	9	45 (20)	8	52 (15)
No	328	47 (24)	232	57 (23)	162	62 (24)
**Obesity (BMI > 35 kg/m²)**						
Yes	11	67 (23)	9	69 (22)	6	75 (29)
No	331	46 (24)	235	56 (23)	166	60 (24)
**Acute Infection**						
Yes	26	37 (19)	16	60 (22)	12	64 (22)
No	309	48 (24)	222	56 (23)	156	61 (24)
**Bedridden**						
Yes	50	37 (24)	30	56 (23)	23	55 (27)
No	284	48 (24)	208	56 (23)	145	62 (23)
**Central venous catheter**						
Yes	198	49 (23)	141	56 (22)	96	61 (23)
No	142	44 (25)	100	56 (23)	73	61 (25)
**Symptomatic VTE**						
Yes	262	45 (25)	183	56 (23)	132	62 (24)
No	87	52 (21)	65	57 (24)	44	59 (22)
**Superficial VTE**						
Yes	19	49 (27)	13	63 (24)	13	72 (22)
No	332	47 (24)	237	56 (23)	165	60 (24)
**Deep VTE**						
Yes	249	47 (23)	178	55 (23)	129	59 (24)
No	102	47 (26)	72	59 (23)	49	65 (22)
**Bilateral VTE**						
Yes	20	42 (19)	11	46 (25)	8	50 (27)
No	331	47 (24)	239	57 (23)	170	61 (24)
**Pulmonary embolism**						
Yes	116	45 (26)	82	57 (23)	55	62 (19)
No	235	48 (23)	168	56 (23)	123	60 (26)
**CVC-related thrombosis**						
Yes	29	57 (18)	22	63 (20)	11	67 (21)
No	322	46 (24)	228	56 (23)	167	60 (24)
